# Frequency, Plausibility and Internal Consistency of International Classification of Diseases (ICD) Coding Related to Cystic Echinococcosis Within the Jordanian Ministry of Health Database: A Quality-Assurance Study

**DOI:** 10.3390/healthcare14162477

**Published:** 2026-08-11

**Authors:** Shifaa’ Al Qa’qa’, Amal Abu Omar, Abdellateef Abdelhafez Alqawasmi, Khairat Battah, Yamamah Al-Hmaid, Raya Marji, Mais Alkhalili, Dima Hamarsheh, Silvia D. Boyajian, Ensaf Y. Almomani, Lama Hamadneh, Ayssar Tashtush

**Affiliations:** 1 Department of Pathology and Forensic Medicine, Faculty of Medicine, Al-Balqa Applied University, Al-Salt 19117, Jordan; kh.battah@bau.edu.jo (K.B.); r.marji@bau.edu.jo (R.M.); 2 Department of Physiology and Biochemistry, Faculty of Medicine, Jordan University of Science and Technology, Irbid 22110, Jordan; aaabuomar8@just.edu.jo (A.A.O.); aatashtush@just.edu.jo (A.T.); 3 College of Education, Humanities and Social Sciences, Al Ain University, Al Ain 64141, United Arab Emirates; abdellateef.alqawasmi@aau.ac.ae; 4 Department of Public Health and Community Medicine, Faculty of Medicine, Al-Balqa Applied University, Al-Salt 19117, Jordan; y.alhmaid@bau.edu.jo (Y.A.-H.); m.alkhalili@bau.edu.jo (M.A.); 5 Department of Basic Medical Sciences, Faculty of Medicine, Al-Balqa Applied University, Al-Salt 19117, Jordan; d.hamarsheh@bau.edu.jo (D.H.); silvia.boyajian@bau.edu.jo (S.D.B.); ensaf.momani@bau.edu.jo (E.Y.A.); lama.hamadneh@bau.edu.jo (L.H.)

**Keywords:** cystic echinococcosis, Hakeem, health informatics, quality assurance, database, ICD codes, Jordan

## Abstract

**Background/Objectives:** Cystic echinococcosis (CE) remains endemic in many regions worldwide, including Jordan. It is one of the three zoonotic infections caused by the tapeworm of the genus *Echinococcus*. The epidemiology of CE in Jordan remains poorly defined, with systematic national investigations being absent for the past three decades. International Classification of Diseases (ICD) is a universal coding system for disease archiving within healthcare facilities. This coding system is utilized by the Hakeem program, a health informatics system that includes all Ministry of Health (MOH) hospitals and medical centers in Jordan. In this study, we assessed the frequency, plausibility, internal consistency, and administrative limitations of CE-related ICD codes within the MOH database, which have never been tested, and determined the validity of using the administrative data for epidemiological studies of CE in the future. **Methods**: A retrospective database study was conducted, using 12 echinococcosis-related ICD codes for electronic health record review. Data were retrieved from the Hakeem program. Unique patient entries recorded between 2010 and 2024 were extracted, and descriptive analytical methods were applied. **Results**: Over a 15-year period, 45,702 echinococcosis-related entries were reported across 178 healthcare facilities, with Amman accounting for the majority (59.1%) of reported entries. Entry numbers rose in the first 10 years (2010–2019) before a measurable decline and fluctuations in the following 5 years (2020–2024). Entries spanned all age groups, with middle-aged adults being the most frequently reported age group (42.45%). Females accounted for 74.4% of entries, reflecting a marked female predominance among coded entries. The most commonly reported ICD code was B67.8 (unspecified echinococcosis of the liver, 64.22%), followed by B67.5 (*Echinococcus multilocularis* infection of the liver, 33.57%). Confirmatory diagnostic data could not be extracted from the coding system. As a result, clinical validation could not be performed. **Conclusions**: The high frequency of echinococcosis-related entries suggests artificial inflation of CE-related codes, along with other data anomalies, reflecting internal inconsistencies and epidemiological implausibility of the administrative data within the Hakeem program. MOH should implement a dual, system-user-level plan to improve CE-related coding in the future.

## 1. Introduction

Echinococcosis is a globally distributed zoonosis caused by the larval stage of the *Echinococcus* tapeworms [1]. Echinococcosis was historically referred to by several terms, such as hydatid disease and hydatidosis. The World Association of Echinococcosis (WAE) later reached a formal consensus, establishing three standardized terms for diseases caused by the genus *Echinococcus*: cystic echinococcosis (CE), alveolar echinococcosis (AE), and neotropical echinococcosis (NE) [2]. Furthermore, the WAE reclassified the genus *Echinococcus* into nine distinct species and eight genotypes based on molecular testing: *E. granulosus sensu lato* cluster (which causes CE and encompasses *E. granulosus sensu stricto* (*s.s*.) (G1, G3)), *E. equinus* (G4), *E. ortleppi* (G5), *E. canadensis* (G6, G7, G8, G10), and *E. felidis*, in addition to *E. multilocularis* and *E. shiquicus,* which cause AE, and *E. vogeli* and *E. oligarthrus*, which cause NE [2,3].

Of the three aforementioned diseases caused by the genus *Echinococcus*, CE is the most prevalent and widely distributed, reaching endemic levels in Jordan and the Middle East [1]. It is characterized by the development of well-defined cystic lesions, most frequently involving the liver and lungs, though other organs can be affected [4]. While CE can affect all age groups, its incidence increases with age, and it appears to be more common in females [5]. Hydatid cyst development progresses slowly, so patients may remain asymptomatic for several years. In addition, clinical features depend primarily on the cyst’s anatomic site and maximum diameter, leaving diagnosis largely dependent on the circumstances rather than a specific clinical presentation. The clinical picture of this disease takes a dangerous turn when anaphylaxis develops secondary to cyst rupture, which is usually life-threatening [6]. The treatment options for CE include a conservative approach (watch-and-wait), surgical intervention (minimally invasive percutaneous Puncture, Aspiration, Injection, Re-aspiration (PAIR) technique or open or laparoscopic surgical removal), antihelminthic medications, primarily mebendazole and alternatively albendazole, and combined therapy of both surgical and medical treatment, depending on the patient’s circumstances [7,8]. While mebendazole and albendazole are utilized for CE, their indication extends to other helminthic infections [9].

The epidemiology of CE in Jordan remains poorly defined. The available local data are scarce and derived from small retrospective studies, which are confined to geographically specific areas or hospitals, with systematic national investigations being absent for the past three decades [10,11,12,13,14,15,16,17,18,19,20,21,22,23,24,25,26,27,28,29,30,31,32]. The International Classification of Diseases (ICD) is a universal coding system that classifies medical conditions using standard letters and numbers across healthcare systems; these short codes replace longer medical condition nomenclatures. ICD-1 was the first version, established in 1948, and later revised by the World Health Organization (WHO) into the current version, ICD-11, issued in 2019. The primary purpose of the coding system is to archive medical conditions in an organized, easily accessible way for billing purposes, hospital management, and workflow, in addition to research and epidemiological surveillance [33,34]. We launched this retrospective database study to assess the frequency of CE-related ICD codes within the Jordanian Ministry of Health (MOH) database over a 15-year period. In addition, we aimed to assess the plausibility, internal consistency, and administrative limitations of CE-related ICD code entry, which has never been tested before, to determine the validity of employing the administrative data to address the epidemiological gap of CE in Jordan in the future. We utilized the Electronic Health Solutions (EHS) Hakeem program, a health informatics system, which was introduced in Jordan toward the end of 2009 [35].

## 2. Materials and Methods

We used a retrospective administrative database quality-assurance study to examine CE-related entries within the Jordanian MOH for the 15-year period between 2010 and 2024. Eligibility was ascertained via an electronic health record review using the Hakeem program database (Health Electronic Solutions, Amman, Jordan; https://ehs.com.jo/electronic-health-solutions-ehs, accessed on 16 January 2026). Following ethical approval from the Research Ethics Board of Al-Balqa Applied University (2024/2023/3/21) and the Research Ethics Committee (REC) of MOH (MOH/REC/2024/173), anonymized data extraction was performed between January 2025 and April 2025 by the Hakeem program technical team, encompassing MOH hospitals and health centers across Jordan’s three regions (Northern Region, Southern Region, and Central Region). Reporting of code algorithms and data cleaning methodology is precluded as the authors have no access to these data.

**Figure 1:**
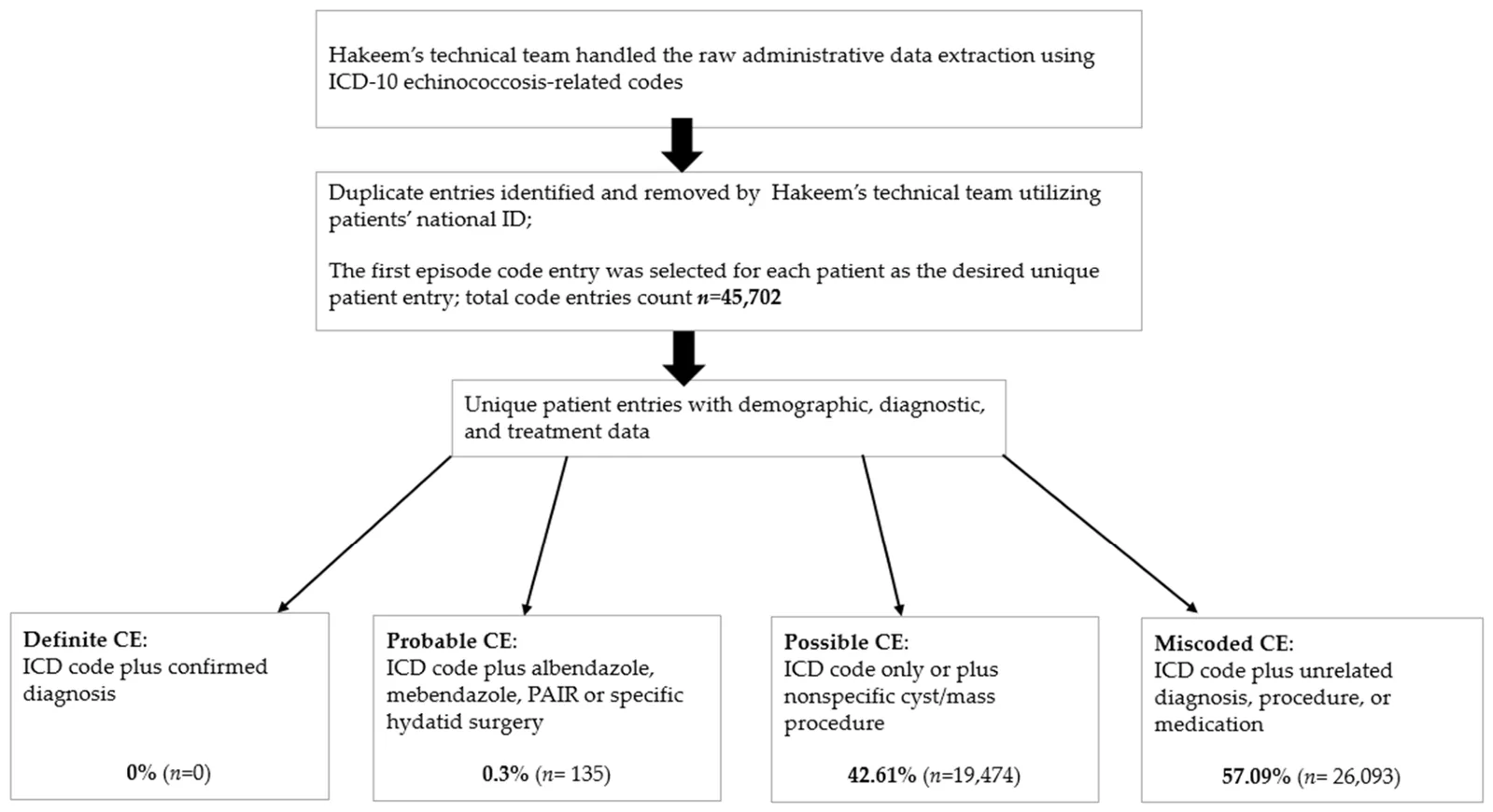
Flow diagram of data selection pathway: The total number of entries depends on the index encounter coding, which reflects unique patient entries that were finally categorized into four groups: definite CE, probable CE, possible CE, and miscoded CE.

Identification of eligible entries relied on the ICD codes, version 10 (ICD-10). ICD-11 has not yet been adopted by the EHS Hakeem program throughout the study period. The search strategy included codes related to *E. granulosus* species—B67.0, B67.1, B67.2, B67.31, and B67.4—which referred to infection of the liver, lungs, bone, thyroid gland, and unspecified location, respectively. Codes for *E. multilocularis* species were also used, namely, B67.5, B67.61, B67.69, and B67.7, which related to infection of the liver, multiple sites, other sites, and unspecified location, respectively. Lastly, three other codes were used: B67.8 (echinococcosis, unspecified, of the liver), B67.90 (echinococcosis, unspecified), and B67.99 (other echinococcosis). The Hakeem technical team performed raw data extraction, including all entries related to echinococcosis generated during the assigned years. Notably, duplicate entries from repeated encounters/visits/admissions for the same patient were identified through the patients’ unique national identification (ID) numbers; only the first-time entry that could be a billing encounter, follow-up visit, problem-list entry, or admission entry was counted for data selection. The final code entries indicate unique patient entries. Demographic and treatment data were also extracted (Figure 1).

In more detail, the following data points were requested: entry count, age, sex, nationality, anatomical site, health facility location, diagnostic modality (clinical, radiological, pathological, serological, and molecular findings), and treatment (medications and surgical procedures). Data were compiled using a spreadsheet software tool (Microsoft Excel, v16.0, Microsoft Corp., Redmond, WA, USA) and subsequently analyzed using descriptive analytical methods, using the Microsoft Excel filtering features, to figure out the frequency, annual trend, regional distribution, demographic features, and data validity of the entries. Cross-referencing of ICD codes with confirmed diagnosis, surgical procedures and medications was performed to categorize the unique patient entries into four groups as follows (Figure 1):

1. Definite CE: CE-related code plus confirmed CE diagnosis via surgical, clinical, pathologic, radiologic, serologic, or molecular report data.
2. Probable CE: CE-related code plus antihelminthic and/or surgical procedure related to CE.
3. Possible CE: CE-related code only or plus non-specific cyst/mass surgical procedure.
4. Miscoded CE: CE-related code plus unrelated diagnosis, medication or surgical procedure.

## 3. Results

### 3.1. Frequency of Codes

A total of 45,702 unique patient entries related to echinococcosis were recorded from 178 health facilities, reflecting the high frequency of CE-related code usage. Among the entries, B67.8 (echinococcosis, unspecified, of the liver) was the most utilized ICD code, accounting for 64.22% of entries (*n* = 29,352), followed by B67.5 (*E. multilocularis* infection of liver) at 33.57% (*n* = 15,342) (Table 1). Regarding *Echinococcus* species, unspecified echinococcosis represented the largest proportion of entries, constituting 65.22% (*n* = 29,809). Notably, *E. multilocularis* accounted for 34.65% (*n* = 15,836), and *E. granulosus* remained rare, at just 0.12% (*n* = 57). Regarding anatomical site, hepatic site showed a marked predominance, comprising 97.81% of entries (*n* = 44,699). Other anatomical sites were considerably less frequent and included multiple sites (0.64%, *n* = 294), the thyroid gland (0.06%, *n* = 29), lung (0.04%, *n* = 18), and bone (0.01%, *n* = 3). Additionally, a considerable number of entries (1.44%, *n* = 659) lacked anatomical site specification.

### 3.2. Annual Trend and Regional Distribution

**Figure 2:**
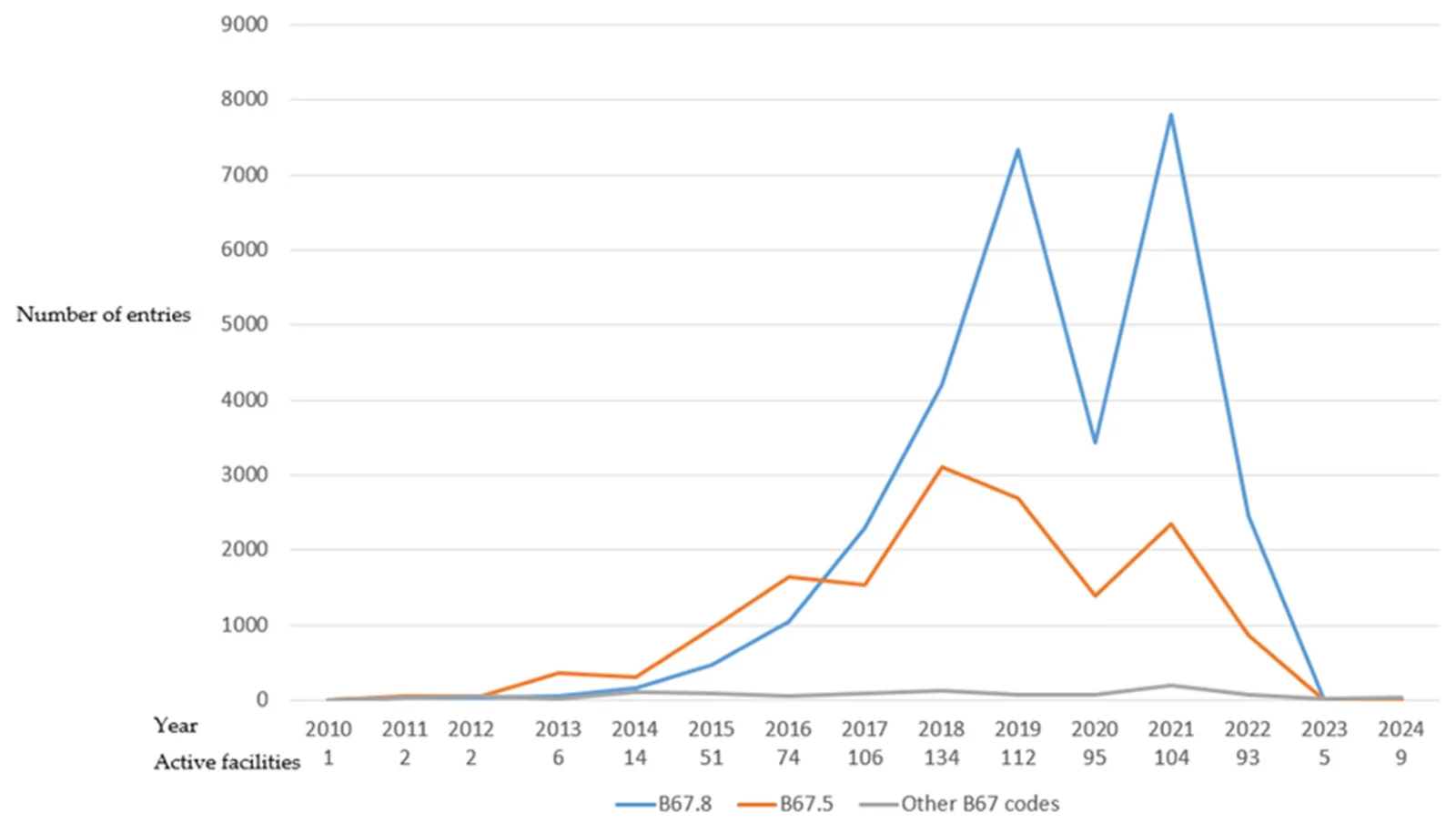
Annual distribution and active health facilities: Three code groups were defined, including B67.8, B67.5, and other B67 codes. The number of entries showed an increase in the first 10 years (2010–2019), then declined and fluctuated over the last 5 years (2020–2024).

**Figure 3:**
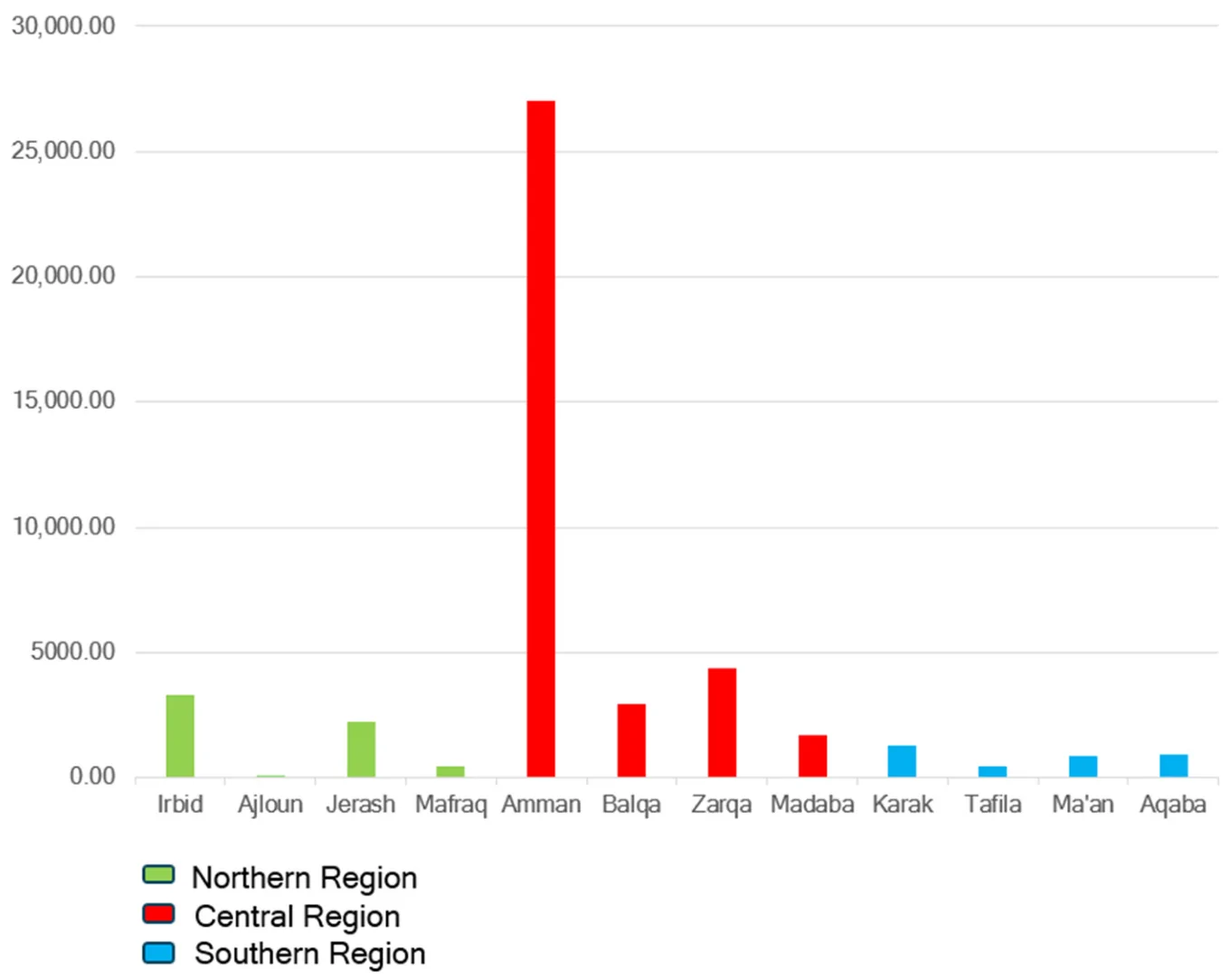
Regional distribution of entries: The vast majority of entries originated from the Central Region (78.88%). The Northern and Southern Regions showed lesser contributions.

An observed increase in entries occurred between 2010 and 2019, the first 10 years of the study period, after which the numbers declined and fluctuated between 2020 and 2024, the last 5 years of the study period (Figure 2). Peak values were noted in 2018, 2019, and 2021. The entries spanned all of Jordan’s 12 governorates across three geographical regions (Table 2, Figure 3), with a clear concentration in the Central Region (78.88%, *n* = 36,049), followed by the Northern Region (13.47%, *n* = 6156) and the Southern Region (7.65%, *n* = 3497). In more detail, the majority of entries were concentrated in Amman (59.09%, *n* = 27,004), followed by Zarqa (9.62%, *n* = 4397), Irbid (7.27%, *n* = 3322), Balqa (6.40%, *n* = 2926), Jerash (4.95%, *n* = 2264), Madaba (3.77%, *n* = 1722), Karak (2.78%, *n* = 1271), Aqaba (2.03%, *n* = 929), Ma’an (1.86%, *n* = 848), Mafraq (1.07%, *n* = 487), Tafila (0.98%, *n* = 449), and Ajloun (0.18%, *n* = 83). Notably, many governorates reported no entries for multiple years, particularly in the earlier years of the study period.

### 3.3. Demographic Features

The demographic distribution of entries (Table 3) reveals a marked female predominance (74.4%, *n* = 33,996). Ages range from 0 to 97 years and are categorized into four age groups, as follows: children (16.45%), young adults (30.62%), middle-aged adults (42.45%), and older adults (10.48%). Notably, 282 entries (0.62%) were recorded for infants (under the age of 1). A total of 854 entries (1.87%) were recorded among residents of non-Jordanian nationality.

### 3.4. Administrative Data Validity and Unique Patient Entry Categories

Confirmatory data, including clinical, radiological, pathological, serological, and molecular findings, could not be extracted from the Hakeem program, as these data were presented in unformatted note sheets. As a result, validation of entries as true CE could not be performed with certainty, reflecting administrative data limitations.

Surgical procedures were recorded for 4124 entries, whereas medications were recorded for 22,179 entries (Table 4). Recorded treatment, including surgeries and medications, was widely diverse, and most of which were unrelated, such as dilation and curettage (D&C) and proton pump inhibitors (PPIs), or non-specific, such as cyst/mass excision. Relevant and more specific surgical procedures were found for only 17 entries, including craniotomy for hydatid cyst, deroofing of hydatid cyst, deroofing of multiple hydatid cysts, external drainage and deroofing of hydatid cyst of liver, hydatid cyst, hydatid cyst excision, hydatid liver cyst, laparoscopic deroofing of hydatid liver cyst, laparoscopic liver hydatid cystectomy and renal cystectomy, laparoscopic pericystectomy of hydatid cyst, lung hydatid cyst excision, and thoracotomy for hydatid cyst. Antihelminthic medications (albendazole or mebendazole) were recorded for 118 entries.

Accordingly, the percentage and number of unique patient entries for the previously defined categories are as follows (Figure 1): 0% definite CE (*n* = 0), 0.3% probable CE (*n* = 135), 42.61% possible CE (*n* = 19,474) and 57.09% miscoded CE (*n* = 26,093).

## 4. Discussion

We utilized the ICD coding system throughout the Hakeem program database to assess the frequency, plausibility, internal consistency, and administrative data limitations of entries related to CE at MOH hospitals and medical centers in Jordan. We found a substantial number of echinococcosis-related ICD codes in the MOH database, totaling 45,702 unique patient entries, including mostly non-specific echinococcosis-related entries, as well as AE (*E. multilocularis*)- and CE (*E. granulosus*)-related entries, and the liver showed the predominant anatomical site. The number of entries showed fluctuations over the assigned years and a clear concentration in the Central Region. Entries were recorded for patients between the ages of 0 and 97 years, and predominantly in females. Confirmatory diagnostic data retrieval via the Hakeem program could not be achieved, which represented the main limitation of the administrative data, which remained unverified. The overall data anomalies, including artificial data inflation, concentration of entries in Amman, zero entries in many governorates for more than one year, and fluctuations in entry numbers among years and regions, indicate internal inconsistency and implausibility of CE-related codes, limiting future epidemiological studies.

A total of 45,702 entries over 15 years were found in our study, a considerable number compared with previous local studies, with unspecified echinococcosis representing the largest proportion of entries (65.22%), followed by *E. multilocularis* (34.65%), and *E. granulosus* (0.12%) species. Meanwhile, previous studies documented only 2338 cases of CE in Jordan between 1966 and 2015 [10,11,12,13,14,15,16,17,18,19,20,21,22,23,24,31,32]. Confirmatory diagnostic data are lacking in this study, with zero entries of definite CE, only 135 entries of probable CE and 19,474 entries of possible CE. Most entries (26,093) are considered miscoded CE. These hypothesized categories were mostly inferred from cross-referencing code entries with administrative treatment data. However, treatment is neither a specific nor a sensitive diagnostic reference standard for CE, which can be managed conservatively or by surgery or medical treatment or a combined approach depending on the patient’s circumstances [7,8]. Further, albendazole and mebendazole can be used for other helminthic infections [9], and surgery may be miscoded or non-specific. This significant discrepancy in the count between the current study and previous local studies suggests implausibility and artificial data inflation of CE-related codes throughout the MOH Hakeem program. This is particularly evident as an internal inconsistency given that most entries are associated with unrelated or non-specific surgical procedures, such as D&C and cyst/mass excision, respectively. CE-related codes may be misused for other unrelated medical conditions, which should also be investigated in the future.

Notably, a significant proportion of entries (34.65%) were for *E. multilocularis* infection, which is reflecting a coding problem, as the *E. multilocularis* species has never been documented in Jordan; human CE in Jordan has been proven to be caused by *E. granulosus* (*s.s.*), G1 and G3 [29,30]. This coding problem may stem from code-selection errors by Hakeem users, or may indicate software technical issues such as default mapping or poorly configured electronic ICD pick-lists [36,37,38]. This should be investigated to figure out whether the problem is user- or system-dependent; MOH should implement a plan to improve coding selection, such as increasing awareness of CE coding among healthcare workers, including the correct species type.

Our study revealed a significant variation in entry number among regions, with a clear concentration in the Central Region (78.88%), with the majority of entries concentrated in Amman (59.09%), Zarqa (9.62%), and Balqa (6.40%), followed by the Northern Region (13.47%), with most of the entries originating from Irbid (7.27%), and the Southern Region (7.65%), with most contributions originating from Karak (2.78%). Our findings regarding the geographical distribution of entries are not entirely consistent with the literature. A significant study by Kamhawi (1985–1993), spanning 18 major Jordanian hospitals, identified the largest reported human CE cohort in the country (676 cases) and revealed a surgical incidence rate ranging from 0.5% to 8.2% per 100,000 population, with a mean of 2.9% per 100,000. This earlier investigation found that most cases originated from rural Eastern and Southwestern Regions [18]. More recently, Al-Radaideh et al.’s study (2005–2015) analyzed 217 surgically managed CE cases from four major hospitals in Jordan, with the highest proportion of cases (49.7%) originating from the Central Region, followed by the Northern Region (38.2%) and the Southern Region (11.9%), with specific contributions from Al-Mafraq (22.5%), Amman (20.2%), and Al-Balqa (14.7%) governorates [24]. In comparison, our study included fewer entries from rural Eastern (such as Mafraq) and Southern Regions (such as Tafila), which are considered hyperendemic areas in the previous local studies [18,24,28], thus suggesting under-reporting in these regions. Many governorates have had zero entries for many years, especially in earlier years, reflecting the initiation of the Hakeem program in Jordan around the end of 2009 [35]. ICD coding from health centers in geographic areas with fewer coding contributions should be investigated for potential under-reporting.

Most entries in our study were related to liver anatomical site, recorded for all age groups and showed a marked female predominance; these are findings that serve as invalidated descriptive observations rather than true epidemiological evidence of CE. Predominance of liver anatomical site can be explained by the quick-click phenomenon or administrative coding habit by busy or non-clinical users or system-related default mapping errors [36,37,38]. This also explains the marked female predominance, as echinococcosis-related codes are used as an alternative shortcut default option for other liver pathologies, such as simple liver cysts, which are more common in females [39], resulting in artificial inflation of data within females. Furthermore, the disproportionately low percentage of pulmonary anatomical site, the second most common site of CE [4,18,24], suggests that codes for pulmonary CE were significantly underutilized rather than a true epidemiological finding, which could be due to the previously mentioned administrative coding behavior and software technical issues, or it may reflect patient-based first-visit data selection bias in multi-organ CE, where the pulmonary anatomical site was coded on subsequent episodes. Complex cases including multi-organ involvement and atypical presentation [40] cause coding fragmentation between departments and failure of complete data extraction.

The overall data anomalies we observed in our study, such as the use of *E. multilocularis*-related code, regional variation, treatment mismatch, and low pulmonary entry numbers, suggest a possible coding problem within the Hakeem program or data-extraction errors. Hakeem program coding problems can stem from several factors, including user- and program-dependent errors, such as coding performed by non-clinical or busy staff, reimbursement and non-reimbursement coding-related behavior, coding defaults, software mapping errors, poorly defined pick-lists, and initiation of a new coding system. Data-extraction errors may include duplicate episodes and incomplete data extraction, among others [36,37,38,41,42].

A point to be highlighted is that administrative ICD databases are primarily designated for management and billing purposes within healthcare centers, which should never be treated as definite disease registries [36,43]. However, secondary utilization of these databases as disease registries for research and epidemiological studies is an excellent idea. This requires a registry-quality validation step, which can be achieved by designing a health informatics system with features of direct linking of administrative ICD data with confirmatory diagnoses in clinical, pathological, radiological and laboratory systems that fulfill the WHO disease registry criteria [44]. This plan would change the future of health informatics in Jordan and aid in building a nationwide disease registry.

We propose a validation pilot study including 300 randomly selected unique patient entries, reviewing their full medical data reports, and subsequently determining true positives, false positives and false negatives, and calculating positive predictive value, sensitivity, and specificity. This will help in developing standardized coding guidelines, including clear user interface pick-lists, and are subject to the recommendation of WAE for disease terminology and surgical operation description of CE, with risk stratification of patients based on WHO-Informal Working Group on Echinococcosis criteria for CE diagnosis (suspected cases, probable cases and confirmed cases) based on imaging, serologic and histopathologic findings. At present, performing this validation study is precluded by the strict policy of MOH REC regarding access to patients’ national IDs, which might be addressed in future studies.

The main limitation of our study was the inability to retrieve the confirmatory diagnostic data that are present in free-text clinical notes, as the Hakeem program lacks the property of direct linking of administrative ICD data with confirmatory diagnoses in clinical, pathological, radiological and laboratory systems. Further, manual reviewing of electronic health records is precluded by the current ethical policy of the MOH. Absolute reliance on an unverified, administrative database causes a lack of clinical sense and inability to pinpoint the root cause of the observed data anomalies.

## 5. Conclusions

There appear to be notable administrative data anomalies that reflect unreliability and internal inconsistency limiting the utility of unverified CE-related ICD code entries for future epidemiological studies within the MOH Hakeem program. The data likely reveal artificial inflation of CE-related code entries due to possible coding problems. The MOH should implement a dual plan to improve coding within the Hakeem program, targeting both user- and system-related issues.

## Figures and Tables

**Table 1:** The frequency of echinococcosis-related ICD-10 code entries used in the Hakeem program across the MOH (2010–2024).

ICD-10 Code	Interpretation	Number of Entries	Percentage of Entries
B67.8	Echinococcosis, unspecified, of liver	29,352	64.22%
B67.5	*E. multilocularis* infection of liver	15,342	33.57%
B67.61	*E. multilocularis* infection, multiple sites	294	0.64%
B67.90	Echinococcosis, unspecified	229	0.50%
B67.99	Other echinococcosis	228	0.49%
B67.7	*E. multilocularis* infection, unspecified	160	0.35%
B67.69	*E. multilocularis* infection, other sites	40	0.08%
B67.31	*E. granulosus* infection, thyroid gland	29	0.06%
B67.1	*E. granulosus* infection of lung	18	0.03%
B67.0	*E. granulosus* infection of liver	5	0.01%
B67.2	*E. granulosus* infection of bone	3	0.006%
B67.4	*E. granulosus* infection, unspecified	2	0.004%
		Total 45,702	

**Table 2:** Distribution of entries across different regions in Jordan (2010–2024).

Regions							Years									
	2010	2011	2012	2013	2014	2015	2016	2017	2018	2019	2020	2021	2022	2023	2024	Total
**Northern**																
-Irbid	0	0	0	2	17	268	572	402	466	265	190	914	225	0	1	3322
-Ajloun	0	0	0	0	0	0	0	0	7	29	21	23	3	0	0	83
-Jerash	0	0	0	0	0	111	532	642	352	204	53	267	103	0	0	2264
-Mafraq	0	0	0	0	2	24	39	7	171	86	60	72	24	0	2	487
**Central**																
-Amman	7	126	132	334	374	705	832	1906	4364	6874	2980	6220	2112	15	23	27,004
-Balqa	0	0	0	93	149	132	119	115	394	379	329	906	306	1	3	2926
-Zarqa	0	0	0	0	27	215	419	582	687	797	447	889	332	1	1	4397
-Madaba	0	0	0	0	0	33	66	56	307	617	264	291	87	0	1	1722
**Southern**																
-Karak	0	0	0	0	0	0	0	197	553	265	76	139	41	0	0	1271
-Tafila	0	0	0	0	0	0	0	0	0	13	208	193	35	0	0	449
-Ma’an	0	0	0	0	0	17	151	3	70	145	115	275	71	1	0	848
-Aqaba	0	0	0	0	5	22	18	14	53	432	156	167	62	0	0	929
**Total**	7	126	132	429	575	1527	2748	3924	7424	10,106	4899	10,356	3401	18	30	45,702

**Table 3:** Demographic distribution of entries.

	Number of Entries (*n*)	Percentage
Sex		
Female	33,996	74.4%
Male	11,703	25.6%
Age groups (years)		
Children (0–18)	7517	16.45%
Young adults (19–39)	13,993	30.62%
Middle-aged adults (40–64)	19,403	42.45%
Older adults (≥65)	4789	10.48%
Nationality		
Jordanian	44,845	98.13%
Non-Jordanian	854	1.87%

**Table 4:** Cross-referencing treatment with ICD codes.

Treatment	Number of Entries
CE-specific Surgical procedure specific to CE: -Craniotomy for hydatid cyst -Deroofing of hydatid cyst -Deroofing of multiple hydatid cysts -External drainage and deroofing of hydatid cyst of liver -Hydatid cyst -Hydatid cyst excision -Hydatid liver cyst -Laparoscopic deroofing of hydatid liver cyst -Laparoscopic liver hydatid cystectomy and renal cystectomy -Laparoscopic pericystectomy of hydatid cyst -Lung hydatid cyst excision -Thoracotomy for hydatid cyst	*n* = 17
Potentially CE-related Antihelminthic medications: -Albendazole -Mebendazole	*n* = 118
Non-specific Non-specific surgical procedures: e.g., mass/cyst excision	*n* = 75
Unrelated -Surgical procedures unrelated to CE: e.g., D&C (***n*** = 4032) -Medications unrelated to CE: e.g., PPIs (***n*** = 22,061)	*n* = 26,093

## Data Availability

The raw data supporting the conclusions of this article will be made available by the authors on request.

## Abbreviations

The following abbreviations are used in this manuscript:

WAE	World Association of Echinococcosis
CE	Cystic echinococcosis
AE	Alveolar echinococcosis
NE	Neotropical echinococcosis
PAIR	Puncture, Aspiration, Injection, Re-aspiration
ICD	International Classification of Diseases
WHO	World Health Organization
MOH	Ministry of Health
EHS	Electronic Health Solutions
REC	Research Ethics Committee
ID	Identification
D&C	Dilation and curettage
PPIs	Proton pump inhibitors
